# Controlling the Connected Vehicle with Bi-Directional Information: Improved Car-Following Models and Stability Analysis

**DOI:** 10.3390/s21248322

**Published:** 2021-12-13

**Authors:** Ziwei Yi, Wenqi Lu, Xu Qu, Linheng Li, Peipei Mao, Bin Ran

**Affiliations:** 1 School of Transportation, Southeast University, Nanjing 211189, China; yiziwei@seu.edu.cn (Z.Y.); 230198694@seu.edu.cn (W.L.); leelinheng@seu.edu.cn (L.L.); maopeipei@seu.edu.cn (P.M.); bran@seu.edu.cn (B.R.); 2 Jiangsu Province Collaborative Innovation Center of Modern Urban Traffic Technologies, Southeast University, Nanjing 211189, China

**Keywords:** connected vehicle, bidirectional vehicles information structure, acceleration, derived multiple vehicles information structure, linear stability

## Abstract

Connected vehicle (CV) technologies are changing the form of traditional traffic models. In the CV driving environment, abundant and accurate information is available to vehicles, promoting the development of control strategies and models. Under these circumstances, this paper proposes a bidirectional vehicles information structure (BDVIS) by making use of the acceleration information of one preceding vehicle and one following vehicle to improve the car-following models. Then, we deduced the derived multiple vehicles information structure (DMVIS), including historical movement information of multiple vehicles, without the acceleration information. Next, the paper embeds the four kinds of basic car-following models into the framework to investigate the stability condition of two structures under the small perturbation of traffic flow and explored traffic response properties with different proportions of forward-looking or backward-looking terms. Under the open boundary condition, simulations on a single lane are conducted to validate the theoretical analysis. The results indicated that BDVIS and the DMVIS perform better than the original car-following model in improving the traffic flow stability, but that they have their own advantages for differently positioned vehicles in the platoon. Moreover, increasing the proportions of the preceding and following vehicles presents a benefit to stability, but if traffic is stable, an increase in any of the parameters would extend the influence time, which reveals that neither *β*_1_ or *β*_2_ is the biggest the best for the traffic.

## 1. Introduction

Traffic flow modeling is a significant part of traffic flow theory and existing models include microscopic models, mesoscopic models, and macroscopic models [1]. In recent years, the development of advanced technologies such as sensing technology or communication technology enhance the vehicles’ ability to collect information, calculate data, and optimize motion. Under the intelligent connected environment, connected vehicles (CV) could exchange information effectively, and make decisions cooperatively [2,3]. Thus, many extended models based on the traditional models have been proposed to describe the movement of CV, including the bidirectional-looking model and the forward-looking model. Specifically, the bidirectional-looking models utilize information from preceding and following vehicles, and the forward-looking models utilize the information only from preceding vehicles. Both of them contain macroscopic extended forms and microscopic extended forms [4,5,6,7,8,9].

The microscopic extended models include the car-following model [10,11,12,13], and cellular automaton (CA) models [14]. The extended car-following model can be grouped into three categories: extended basic car-following models with specific items (BE-CF), multi-anticipative forward-looking car-following models (MFL-CF), bidirectional-looking car-following models (BL-CF). For BE-CF models, the optimal acceleration/velocity of the target vehicle was affected by one preceding vehicle. While under the structure of MFL-CF models, the optimal acceleration/velocity of the target vehicle was affected by more than one preceding vehicle. In terms of the BL-CF models, not only the vehicles in front but also the vehicles behind were considered. In all, the main distinction between the BE-CF models and the MFL-CF/BL-CF models is that the BE-CF models focus on the movement of the target vehicle and its nearest preceding vehicle. For the target vehicle, its information comes from one preceding vehicle. For the MFL-CF/BL-CF models, at least three vehicles’ information was calculated.

The first BL-CF model was proposed by Herman et al. [15] based on the relative velocity (RV) model, and it employs the information that comes from the preceding vehicle and a following vehicle near the target vehicle. The relative velocity among them was considered and the stability condition of the BL-CF model was investigated. The result shows that the stability improves with the increase in the proportional of both the preceding and following vehicles. After that, researchers studied the different forms of extended models based on the optimal velocity (OV) model [16,17,18,19,20,21,22,23], full velocity difference (FVD) model [24,25,26,27], Gipps’ safe distance model [28], and Gazis–Herman–Rothery (GHR) model [29], intelligent driver model (IDM) [30,31], adaptive cruise control (ACC) model [32]. Among these models, the extended bidirectional OV model and bidirectional FVD model have been studied enthusiastically, while other models have been studied to a relatively lesser extent. In terms of the bidirectional OV model, Nakayama et al. [16] introduced an extended OV model which depends on the headway of the following vehicle, and found that the extension stabilizes the traffic flow. Then, Hasebe et al. [17] expanded this into an arbitrary number of preceding and following vehicles. After that, Ge et al. [18] proposed an extended OV model with the consideration of the arbitrary number of vehicles ahead and one vehicle following. All of them consider the headway of vehicles from extra information. Based on these models, Yang [19] extended the OV model with information of an arbitrary number of preceding vehicles and one following vehicle, considering headways and relative velocities. Hu et al. [21] proposed the extended OV models in considering of distance and relative velocity of multiple vehicles. Zhang et al. [22] explored the distance and relative velocity of multiple vehicles with anticipation time. Studies of bidirectional FVD models are the extension of OV models. Sun et al. [20] extended the bidirectional FVD with headways and relative velocities. Based on Sun, Ma et al. [26] improved the bidirectional FVD model considering relative velocities with reaction-time delay. Hou et al. [24] and Ma et al. [27] explored the influence of driver’s visual angle. Chen et al. [25] extended the reference [20] by considering driver’s velocity difference memory. Moreover, there is also a batch of other extended models. Yang et al. [28] explored the safe distance of the bidirectional-looking model based on Gipps’ safe distance model. Barrachina et al. [29] explored the chaos of bidirectional-looking vehicles based on the GHR model. Wang et al. [32] extended the Adaptive Cruise Control (ACC) system considering the relative velocity and distance of arbitrary vehicles ahead and followed. Yi et al. [33] proposed bidirectional IDM which considered the ratio of back looking distance and desired distance. Montanino et al. [30,31] extended the bidirectional IDM by utilizing the information in the same form as the original model, and explored the pure and mixed traffic flow stability.

These models contain the investigation of the effect of one vehicle preceding and one vehicle following, multiple vehicles preceding and one vehicle following, an arbitrary number of preceding vehicles and one following vehicle, an arbitrary number of vehicles preceding or following vehicles. In addition, the bidirectional-looking information is mainly about velocity difference, time-delay, space headway, or other forms, such as driver’s sensory memory, driver’s visual angle, and desired speed. The BL-CF models based on other forms of CF models are also investigated. For instance, Li et al. [34] proposed a spring-mass system theory with considering the space headway and velocity difference between the host vehicle and its preceding/following vehicles and found out the stability condition as well. Peter et al. [35] presented a bidirectional control framework to describe the general back-looking models. Based on the synchronization theory of complex networks, Du et al. [36] investigated the stability of an extended car-following model under a honk environment.

The existing BL-CF model explores various information based on the bidirectional-looking effect, such as velocity difference, space headway, driver’s visual angle, and desired speed. Among the existing research, the application of the acceleration of the preceding and following vehicles has not been fully studied and discussed. Acceleration represents a decision signal of the vehicle, affecting the next time state of the vehicle. In some models, acceleration is used to represent the longitude decision of a vehicle, such as IDM. When the acceleration information is combined with the motion state of the object vehicle, some information can be obtained indirectly, such as speed and position. Therefore, there is some indirect information contained in the acceleration. If a vehicle’s acceleration can be collected and transmitted to other vehicles through V2V or V2I technology, this information can be used to improve vehicle decision-making in establishing extended car-following models. Li et al. [37] found that the immediate acceleration of preceding vehicles brings about positive effects on traffic flow stability under the MFL-CF structure. While they defined the immediate acceleration of the front vehicle as to the original model of the front vehicle at the last time. Following that logic, the front vehicle does not obtain the acceleration information of the vehicle in front of it. Thus, this case degenerates as the objective vehicle could only receive the velocity, headway, and velocity difference between itself and its nearest preceding vehicle. If the vehicle is homogeneous and each vehicle can be optimized according to the acceleration of the front vehicle when making decisions at each time, the acceleration information of the front vehicle at a certain time contains the historical motion state information of multiple vehicles. Existing research does not dig into the implicit information of acceleration.

In addition, the existing research lacks a comparison between the forward and backward information. In the macroscopic extended traffic flow model, researchers discussed the influence of bidirectional information on anisotropy [1]. Since the traffic flow has a direction, the forward and backward information has different impacts on the traffic flow in the traditional sense. In the microscopic traffic flow, few researchers discuss the difference influence between the forward and backward information on the traffic flow.

The innovation of this paper is to establish a bidirectional acceleration information framework by exploring the relationship between the bidirectional structure and the historical multiple vehicle motion information, deeply understanding the implication of acceleration information, and exploring the difference between the preceding term and the following term. Firstly, we propose a bidirectional vehicles information structure (BDVIS) by making use of the acceleration information of one preceding vehicle and one following vehicle. Then, we deduce the derived multiple vehicles information structure (DMVIS). Next, we embed the IDM, the FVD, the OV, and ACC model into the framework to verify the effects of two structures, investigating the stability condition for traffic flow under the small perturbation and exploring traffic response properties with different proportions of forward-looking or backward-looking terms. In this paper, the linear stability theory [38] is employed to investigate the string stability of traffic flow.

The rest of the paper is organized as follows: Section 2 presents the BDVIS and deduces the DMVIS. The linear stability condition is derived using the Laplace transform-based method, exploring the stability condition of the two structures. Section 3 conducts the numerical simulation to verify the linear stability condition, the disturbance influence time was defined and the vehicle response properties were analyzed. Section 4 is the result and discussion, Section 5 is the conclusion.

## 2. Methodology

### 2.1. Bidirectional Information Structure for Car-Following Model

#### 2.1.1. The BDVIS Based on Acceleration

**Figure 1:**
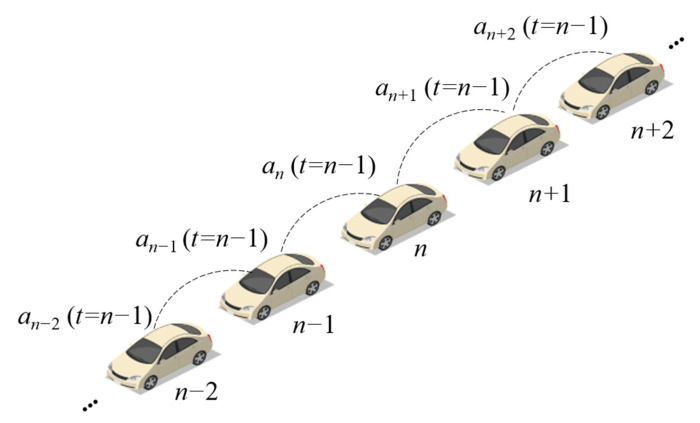
The information exchange between vehicles of the BDVIS.

Under a connected environment, the driver could receive the information of front and rear vehicles, and the acceleration of adjacent vehicles could transmit to the host vehicle. Hence, the acceleration of the near preceding vehicle and the near following vehicle in the last step are considered to optimize the acceleration of the host vehicle. Acceleration represents the tendency of longitudinal movement, which can be utilized to guide the vehicle to adjust its movement in advance. The acceleration information exchange between vehicles is indicated in Figure 1.

The acceleration calculation of the vehicle using the BDVIS is as follows:

(1) $$a_{n}(t)=f(a_{n},t)+\beta_{1}a_{n-1}(t-1)+\beta_{2}a_{n+1}(t-1)$$

where *a_n_*(*t)* is the acceleration of the *n*th vehicle in time step *t*. *f*(*a_n_*, *t*) is the original car-following model, which does not include the acceleration information of vehicles. The parameter *β*_1_ defines the coefficient of the preceding vehicle’s acceleration and the *β*_2_ defines the coefficient of the following vehicle’s acceleration.

Peter et al. [35] found that drivers take the information of the vehicle behind into account, but the main attention of the driver is focused on the preceding vehicle, according to the calibration of the real-world data. The forward information has a greater impact on the vehicle. Therefore, when considering the acceleration of the preceding and following vehicles, the proportion of the preceding vehicle should be larger, that is *β*_1_ > *β*_2_. Since the coefficient of the original car-following model *f(a_n_,t)* is 1, the sum of the coefficient of preceding and the following vehicle should be smaller than the original car-following model, which means *β*_1_ + *β*_2_ < 1.

#### 2.1.2. The DMVIS Deduced from the BDVIS

In the homogeneous traffic flow, all of the vehicles consider the acceleration of their preceding vehicle and following vehicle. The bidirectional acceleration can be deduced from the movement of multiple vehicles as follows:

(2) $$\begin{array}{ll} a_{n}(t) & =f(a_{n},t)+\beta_{1}a_{n-1}(t-1)+\beta_{2}a_{n+1}(t-1)\\ & =f(a_{n},t)+\beta_{1}\left[f(a_{n-1},t-1)+\beta_{1}a_{n-2}(t-2)+\beta_{2}a_{n}(t-2)\right]\\ & +\beta_{2}\left[f(a_{n+1},t-1)+\beta_{1}a_{n}(t-2)+\beta_{2}a_{n+2}(t-2)\right] \end{array}$$

The DMVIS can be deduced by expanding the bidirectional expression, and the acceleration of the *n*th vehicle at time step *t* is expressed as:

(3) $$\begin{array}{ll} a_{n}(t) & =f(a_{n},t)+[\chi_{n-1}\beta_{1}f(a_{n-1},t-1)+\chi_{n+1}\beta_{2}f(a_{n+1},t-1)]\\ & +[\chi_{n-2}\beta_{1}{}^{2}f(a_{n-2},t-2)+2\chi_{n}\beta_{1}\beta_{2}f(a_{n},t-2)+\chi_{n+2}\beta_{2}{}^{2}f(a_{n+2},t-2)]\\ & +[\ldots ]+[\chi_{n-\tau }\beta_{1}{}^{\tau }f(a_{n-\tau },t-\tau )+\chi_{n-\tau +2}\frac{\tau }{1!}\beta_{1}{}^{\tau -1}\beta_{2}f(a_{n-\tau +2},t-\tau )\\ & +\chi_{n-\tau +4}\frac{\tau (\tau -1)}{2!}\beta_{1}{}^{\tau -2}\beta_{2}{}^{2}f(a_{n-\tau +4},t-\tau )+\ldots +\chi_{n+\tau }\frac{\tau !}{\tau !}\beta_{2}{}^{\tau }f(a_{n+\tau },t-\tau )] \end{array}$$

(4) $$\chi_{i}=\{\begin{array}{cc} 1, & 0<i\leq N\\ 0, & others \end{array}$$

where *f*(*a_n_*, *t*) is the original car-following model and *τ* is the time step. *X* is the judgment coefficient to distinguish whether the vehicle is in the platoon, and *N* is the total number of the platoon.

The acceleration of the *n*th vehicle at time *t* is determined by all of the vehicles in the platoon if *τ* is big enough, which can be written as:

(5) $$a_{n}(t)=\sum_{k=0}^{\tau }g(t,k)$$

(6) $$\begin{array}{l} g(t,k)=[m(k,0)\times f(a_{n-k},t-k)+m(k,1)\times f(a_{n-k+2},t-k)\\ \;\;\;\;\;\;\;\;+m(k,2)\times f(a_{n-k+4},t-k)+\ldots +m(k,k)\times f(a_{n+k},t-k)] \end{array}$$

(7) $$m(k,j)=\chi_{n-k+2j}\frac{\prod_{l=k-j+1}^{k}l}{j!}\beta_{1}{}^{k-j}\beta_{2}{}^{j}$$

**Figure 2:**
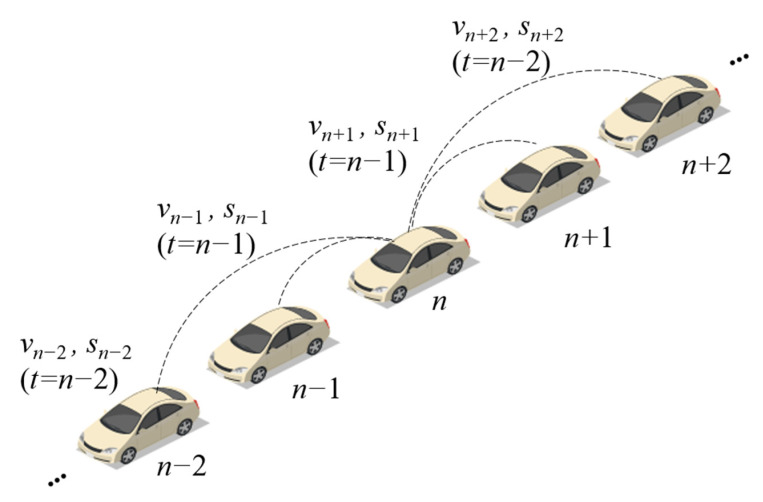
The information access for the nth vehicle in the DMVIS.

Consequently, the information of the host vehicle contains the acceleration of its preceding vehicle in the last time step, the acceleration of its next preceding vehicle in the last two time step, and so on. Similarly, the information of the host vehicle also contains the acceleration of its following vehicle in the last time step, the acceleration of its next following vehicle in the last two time step, and so on. The information access for the *n*th vehicle is shown in Figure 2. The difference between the DMVIS and the BDVIS is that the latter uses the last step of acceleration, but the DMVIS utilizes the multiple steps of information from multiple vehicles.

### 2.2. Linear Stability Analysis for Car-Following Models

In this section, we apply the linear stability method for the two proposed structures. The steady-state has the same homogeneous flow solution, all of the vehicles in the platoon have the same velocity and the same headway.

#### 2.2.1. Stability Analysis for BDVIS

Let *y_n_(t)* be a small perturbation with linear Fourier-mode expanding from the steady-state position of the vehicle *n* at time *t*. The form is as follows:

(8) $$y_{n}(t)=ce^{ia_{k}n+zt}=x_{n}(t)-x_{n}{}^{*}(t)\;\;\;\;y_{n}\left(t\right)\to 0,a_{k}=\frac{2\pi k}{N}\left(k=0,1,L,N-1\right)$$

where *c* is a constant and x_n_^*^(t) is the steady-state of the *n*th vehicle at time step *t*.

The second derivative of both sides of Equation (8) can be conducted:

(9) $${\ddot{y}}_{n}\left(t\right)={\ddot{x}}_{n}\left(t\right)-{\left(x_{n}{}^{*}\left(t\right)\right)}^{\prime \prime }=\frac{dv_{n}(t)}{dt}$$

(10) $$\frac{dv_{n}(t)}{dt}=a_{n}(t)$$

Substituting Equations (8)–(10) into Equation (1):

(11) $$\begin{array}{l} {\dot{y}}_{n}(t+T_{D})-{\dot{y}}_{n}(t)=T_{D}f_{n}^{s}\left(y_{n-1}(t)-y_{n}(t)\right)+f_{n}^{v}\left(y_{n}(t+T_{D})-y_{n}(t)\right)+\\ f_{n}^{\Delta v}\left(y_{n}(t+T_{D})-y_{n}(t)-y_{n-1}(t+T_{D})+y_{n-1}(t)\right)+\beta_{1}({\dot{y}}_{n-1}(t)-{\dot{y}}_{n-1}(t-T_{D}))+\beta_{2}({\dot{y}}_{n+1}(t)-{\dot{y}}_{n+1}(t-T_{D})) \end{array}$$

where

$$f_{n}^{s}=\partial f/\partial s_{n}\left(\bar{s},\bar{v},0\right)\geq 0,f_{n}^{v}=\partial f/\partial v_{n}\left(\bar{s},\bar{v},0\right)\leq 0,f_{n}^{\Delta v}=\partial f/\partial \Delta v_{n,n-1}\left(\bar{s},\bar{v},0\right)\leq 0.$$

*T_D_* is the differential step size. The homogeneous traffic flow satisfies:

(12) $$f_{n}^{s}=f_{n-k}^{s}=\ldots =f_{s},f_{n}^{v}=f_{n-k}^{v}=\ldots =f_{v},f_{n}^{\Delta v}=f_{n-k}^{\Delta v}=\ldots =f_{\Delta v}$$

Substituting Equation (12), $y_{n}(t)=ce^{i\alpha_{k}n+zt}$ and ${\dot{y}}_{n}(t)=zce^{i\alpha_{k}n+zt}$ into Equation (11), the algebraic equation for *z* can be obtained for any mode of perturbation (*k* = 0, 1, …, *n* − 1) as:

(13) $$\begin{array}{l} z\left(e^{zT_{D}}-1-\beta_{1}e^{-i\alpha_{k}}(1-e^{-zT_{D}})-\beta_{2}e^{i\alpha_{k}}(1-e^{-zT_{D}})\right)=T_{D}f_{s}\left(e^{-i\alpha_{k}}-1\right)+\\ \;f_{v}\left(e^{zT_{D}}-1\right)+f_{\Delta v}\left(e^{zT_{D}}-1\right)\left(1-e^{-i\alpha_{k}}\right) \end{array}$$

Expanding $z=z_{1}\left(i\alpha_{k}\right)+z_{2}{\left(i\alpha_{k}\right)}^{2}+\cdots ,e^{z}=1+z+\frac{z^{2}}{2}+\cdots$ and inserting it into Equation (13), the first order and second-order terms of coefficients of the expression of *z* can be given, respectively, as follows:

(14) $$z_{1}=\frac{f_{s}}{f_{v}}$$

(15) $$z_{2}=\frac{f_{s}\left(-\frac{1}{2}f_{v}{}^{2}-\frac{T_{D}}{2}f_{s}f_{v}-f_{v}f_{\Delta v}+f_{s}(1-\beta_{1}-\beta_{2})\right)}{f_{v}{}^{3}}$$

For small disturbances with long wavelengths, the stationary traffic flow is stable if:

(16) $$z_{2}>0$$

When *β*_1_ = 0, *β*_2_ = 0, the stability condition degenerates as the stable condition:

(17) $$z_{2}=\frac{f_{s}\left(-\frac{1}{2}f_{v}{}^{2}-\frac{T_{D}}{2}f_{s}f_{v}-f_{v}f_{\Delta v}+f_{s}\right)}{f_{v}{}^{3}}$$

The traffic flow is stable if *z*_2_ > 0. Under the situation, all vehicles have the same velocity, as a consequence, the acceleration and velocity difference are 0 for all vehicles.

#### 2.2.2. Stability Analysis for DMVIS

Substituting Equations (6), (8)–(10) into Equation (5) can obtain:

(18) $${\ddot{y}}_{n}\left(t\right)=\sum_{k=0}^{\tau }g(t,k)=\sum_{k=0}^{\tau }\begin{array}{l} \left[m(k,0)\times f(a_{n-k},t-k)+m(k,1)\times f(a_{n-k+1},t-k\right)\\ \;+m(k,2)\times f(a_{n-k+4},t-k)+\cdots +m(k,k)\times f(a_{n+k},t-k)] \end{array}$$

Linearizing the $f(a_{n},t)$:

(19) $$\begin{array}{l} T_{D}f_{n}^{s}\left(y_{n-1}(t)-y_{n}(t)\right)+f_{n}^{v}\left(y_{n}(t+T_{D})-y_{n}(t)\right)+\\ \;f_{n}^{\Delta v}\left(y_{n}(t+T_{D})-y_{n}(t)-y_{n-1}(t+T_{D})+y_{n-1}(t)\right) \end{array}$$

where

$$f_{n}^{s}=\partial /\partial s_{n}\left(\bar{s},\bar{v},0\right)\geq 0,f_{n}^{v}=\partial f/\partial v_{n}\left(\bar{s},\bar{v},0\right)\leq 0,f_{n}^{\Delta v}=\partial f/\partial \Delta v_{n,n-1}\left(\bar{s},\bar{v},0\right)\leq 0.$$

Substituting Equations (11) and (19), $y_{n}(t)=ce^{i\alpha_{k}n+zt}$ and ${\dot{y}}_{n}(t)=zce^{i\alpha_{k}n+zt}$ into Equation (18), the linearization of $a_{n}(t)$ can be simplified as:

(20) $$\begin{array}{l} z\left(e^{zT_{D}}-1\right)=\left[T_{D}f_{s}\left(e^{-i\alpha_{k}}-1\right)+f_{v}\left(e^{zT_{D}}-1\right)+f_{\Delta v}\left(e^{zT_{D}}-1\right)\left(1-e^{-i\alpha_{k}}\right)\right]\times \\ \sum_{k=0}^{\tau }e^{-zk}[m(k,0)e^{-i\alpha_{k}k}+m(k,1)e^{-i\alpha_{k}(k-2)}+m(k,2)e^{-i\alpha_{k}(k-4)}+\ldots +m(k,k)e^{-i\alpha_{k}(-k)}] \end{array}$$

Expanding $z=z_{1}\left(i\alpha_{k}\right)+z_{2}{\left(i\alpha_{k}\right)}^{2}+\cdots ,e^{z}=1+z+\frac{z^{2}}{2}+\cdots$ and inserting it into Equation (20), the first order and second-order terms of coefficients of the expression of *z* can be given, respectively, as follows:

(21) $$z_{1}=\frac{f_{s}}{f_{v}}$$

(22) $$z_{2}=\frac{f_{s}\left(-\frac{1}{2}f_{v}{}^{2}\sum_{k=0}^{\tau }\sum_{j=0}^{k}m(k,j)-\frac{T_{D}}{2}f_{s}f_{v}\sum_{k=0}^{\tau }\sum_{j=0}^{k}m(k,j)-f_{v}f_{\Delta v}\sum_{k=0}^{\tau }\sum_{j=0}^{k}m(k,j)+f_{s}\right)}{f_{v}{}^{3}\sum_{k=0}^{\tau }\sum_{j=0}^{k}m(k,j)}$$

#### 2.2.3. Results of Stability Analysis

In this paper, four kinds of car-following models—the OV, FVD, ACC, and IDM—are introduced to analyze the linear stability with the structure of the BDVIS and DMVIS. Table 1 list the formulation and parameters of four classical models.

**Figure 3:**
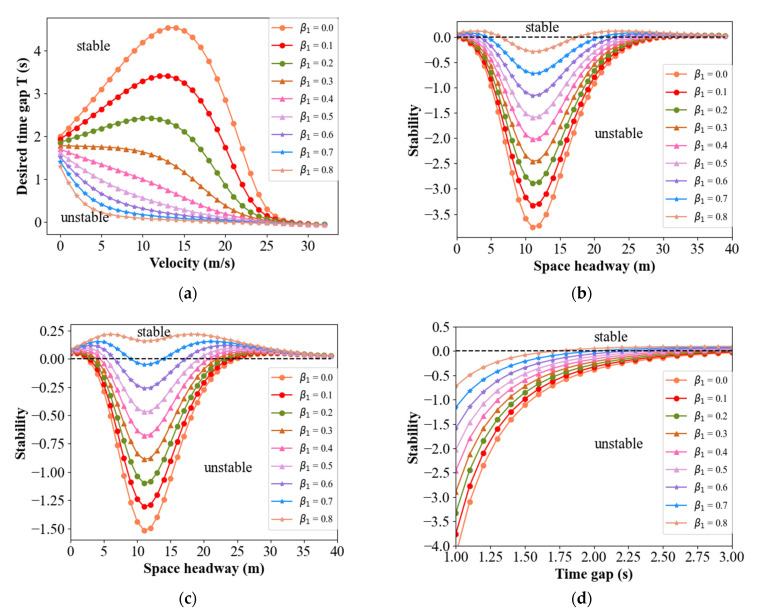
Relationship between the string stability of the traffic and *β*_1_ under the BDVIS for four models. (**a**) Stability curves of traffic in IDM under the BDVIS; (**b**) Stability curves of traffic in FVD under the BDVIS; (**c**) Stability curves of traffic in OV model under the BDVIS; (**d**) Stability curves of traffic in ACC model under the BDVIS.

For small disturbances with long wavelengths, the stationary traffic flow is stable if *z*_2_ > 0. Substituting *T_D_* = 0.1, and the values in Table 1 in Equation (14), the stability regions for four classical models in BDVIS can be explored. It can be found in Equation (14) that *β*_1_ and *β*_2_ have the same effect on the theoretical analysis. Hence, the relationship between traffic stability and parameters *β*_1_ or *β*_2_ can be studied by exploring the change in *β*_1_. Figure 3 shows the relationship between the string stability of the traffic and *β*_1_, under the condition where *β*_2_ = 0.

**Figure 4:**
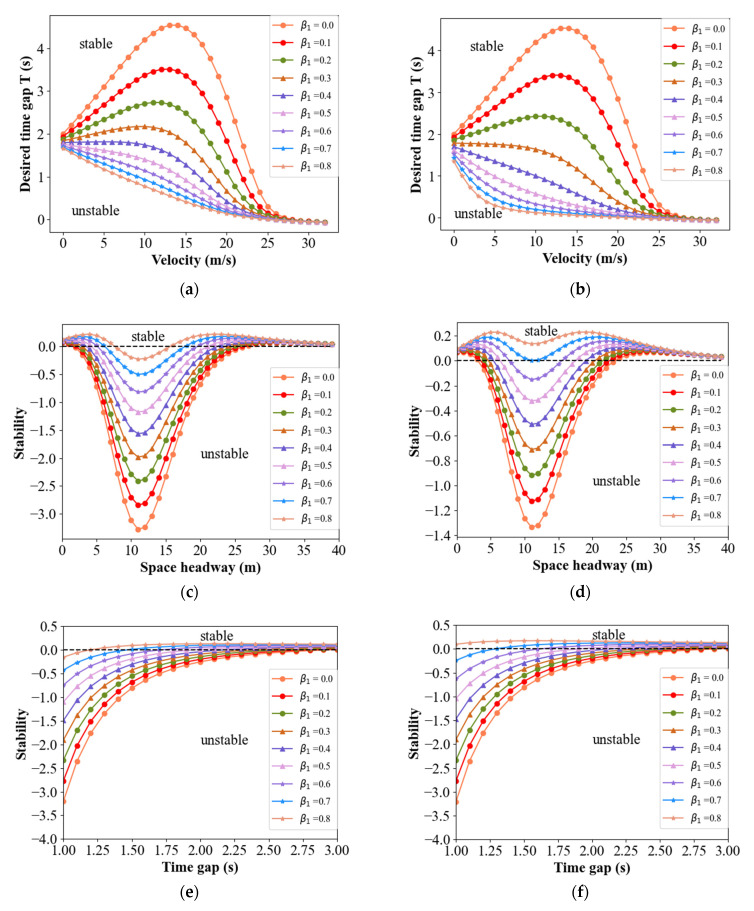
Relationships among the string stability of the traffic, *β*_1_, and the vehicle position under the DMVIS for four models. (**a**) Stability curves for 2nd vehicle in IDM under the DMVIS; (**b**) Stability curves for 8th vehicle in IDM under the DMVIS; (**c**) Stability curves for 5th vehicle in FVD under the DMVIS; (**d**) Stability curves for 5th vehicle in OV model under the DMVIS; (**e**) Stability curves for 5th vehicle in ACC model under the DMVIS; (**f**) Stability curves for 8th vehicle in ACC model under the DMVIS.

**Figure 5:**
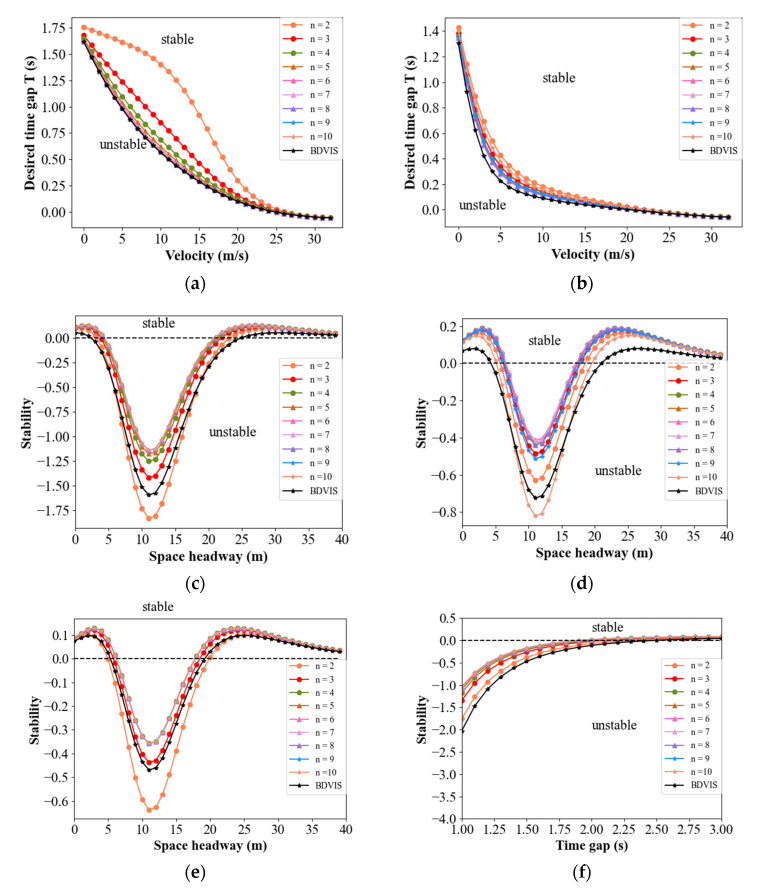
The stability for vehicles under the BDVIS and different positions of the vehicles under the DMVIS. (**a**) Relationship between platoon length and stability in IDM when *β*_1_ = 0.5 and *β*_2_ = 0.1; (**b**) Relationship between platoon length and stability in IDM when *β*_1_ = 0.5 and *β*_2_ = 0.3; (**c**) Relationship between the position of the vehicles and stability in FVD when *β*_1_ = 0.5 and *β*_2_ = 0.0; (**d**) Relationship between the position of the vehicles and stability in FVD when *β*_1_ = 0.4 and *β*_2_ = 0.3; (**e**) Relationship between the position of the vehicles and stability in OV model when *β*_1_ = 0.5 and *β*_2_ = 0.0; (**f**) Relationship between the position of the vehicles and stability in ACC model when *β*_1_ = 0.5 and *β*_2_ = 0.0.

The vertical axis in Figure 3a, Figure 4a,b and Figure 5a,b, represent the desired time gap. These figures display that the traffic remains stable under conditions where the desired time gap is greater than the corresponding value. Other figures, where the vertical axis is stable, display the stability result using Equation (14) or Equation (22). This means that the traffic is stable when the stability results are bigger than 0. Note that the black line is the boundary.

In Figure 3a, the lines decline with the increase in *β*_1_, indicating that the stability region of traffic flow is increased. For Figure 3b–d, the line rises with the increase in *β*_1_, resulting in more cases above the black line. Consequently, the string stability of the traffic flow is enhanced in all four models under the BDVIS. Similarly, the string stability of the traffic flow increases with the increase in *β*_2_. This means that the traffic flow will be more stable by considering the acceleration of the preceding or the following vehicle.

Substituting *T_D_* = 0.1, and the values in Table 1 in Equation (22), the stability regions for four classical models in BDVIS can be explored. It can be found from Equation (22), as well as Equation (14), that *β*_1_ and *β*_2_ are symmetrical on the expression of stability formula. However, the position of vehicles on the platoon affects the information acquirement. Figure 4 shows the relationship among *β*_1_, the vehicle position, and the string stability of the traffic. These cases are carried out in the platoon with 10 vehicles, obeying four models under the DMVIS, respectively.

In the same models under the DMVIS, the stability improves as the vehicle’s position increases. The reason for this is that the host vehicle receives more abundant information when its position is further back. Readers should note that the premise is the backward information is zero. It can be inferred from the symmetry in the formula that the traffic has the same characteristics as the *β*_2_. Moreover, the shape of the stability region in the DMVIS is approaching the stability region in the BDVIS to some degree. This means that the immediate acceleration of the near preceding and following vehicles can be replaced by the movement information of multiple vehicles in some circumstances.

In contrast to the BDVIS, the stability of DMVIS is influenced by the position of the vehicles. Figure 5 compares the stability curves between BDVIS and information from different positions of vehicles under DMVIS in a certain combination of *β*_1_ and *β*_2_. The total number of the platoon equals 10, and *n* denotes the different positions of the vehicles under the DMVIS. It can be seen that vehicles in the rear of the platoon perform better than those in the front of the platoon when *β*_1_ is large and *β*_2_ is relatively small. Vehicles on the two sides of the platoon perform worse than those in the middle section when *β*_1_ is close to *β*_2_. In all, vehicles in the middle of the platoon perform better than those in the front and rear parts in DMVIS. In addition, models with BDVIS perform better than those in DMVIS in some circumstances and sometimes the opposite is true. However, they are close to each other approximately. The theoretical analysis reveals the acceleration implied history movement information of multiple vehicles. However, the degree of implication is limited and they can only replace each other approximately. As a result, both structures have advantages in terms of improving traffic stability. If vehicles are on the two sides of the platoon, choosing the BDVIS is better to improve string stability. If vehicles are in the middle section of the platoon and are able to obtain the multiple steps of historical information, DMVIS is a better option.

## 3. Experiment and Simulation

### 3.1. Description of the Experiments

Table 2 lists several experiments which aim to:

(1) Verify the theoretical stability results through numerical simulations of a platoon, taking the BDVIS as the representative. (Experiments 1)
(2) Explore traffic response properties with different proportions of forward-looking (*β*_1_) or forward-looking terms (*β*_2_) based on the BDVIS. (Experiments 2~3)

Experiments are conducted by numerical simulations of a platoon under the open boundary condition on a single lane.

### 3.2. Evaluation Index

The disturbance influence time (DIT) is defined to investigate the vehicle response properties under the combination of different proportions of the forward-looking term (*β*_1_) or forward-looking term (*β*_2_). The disturbance influence time describes the acceleration fluctuation time influenced by the perturbation for each vehicle, which is the duration of time that the vehicle begins to decelerate or accelerate due to the influence of the perturbation, and then go back to the equilibration so that the acceleration returns to zero and remains zero. The mathematical expression of the assumption is as follows:

(23) $$DIT_{n}={\left(T_{S}-T_{B}\right)}_{n}$$

where *DIT_n_* is the disturbance influence time of the *n*th vehicle. *T_s_* is the time when the vehicle goes back to the equilibration so that the acceleration returns to zero and remains zero. *T_B_* is the time when the vehicle begins to decelerate or accelerate in the influence of the perturbation. (*T_s_* − *T_B_*)*_n_* is the time for the *n*th vehicle.

### 3.3. Simulation Environment

The experiments were implemented in MATLAB. They were conducted on hardware configured with Inter Core i7-10750H CPU, 16GB memory, an Nvidia GeForce GTX 1650 GPU graphics card.

In the proposed structure, the moving of the platoon was simulated for 3500 s. All the vehicles ran at the initial velocity *v_0_* = 10 m/s, other parameters were set as Table 1 and Table 2 indicate. Except for FVD and OV model, the initial space distance ∆*x* = 20 m; other experiments set the initial space distance ∆*x* = 40 m. To eliminate the influence of the initial state, the disturbance was applied after the traffic reached equilibrium. The equilibrium criterion was *a* = 0 m/s^2^ for all the vehicles. The disturbance induced by the leading vehicle suddenly decelerated with *a* = −1 m/s^2^ during the time *t* = 600 s to *t* = 602 s, and *a* = 0 m/s^2^ was maintained after that. Then, the response of the following vehicles was investigated with the different combinations of parameters in the structure. The simulation was divided into two groups distinguished by the number of vehicles in the platoon. To verify the analytical results, the experiment was conducted in a platoon that contains 100 vehicles.

## 4. Result and Discussion

### 4.1. Verification of Theoretical Stability Results of the BDVIS

**Figure 6:**
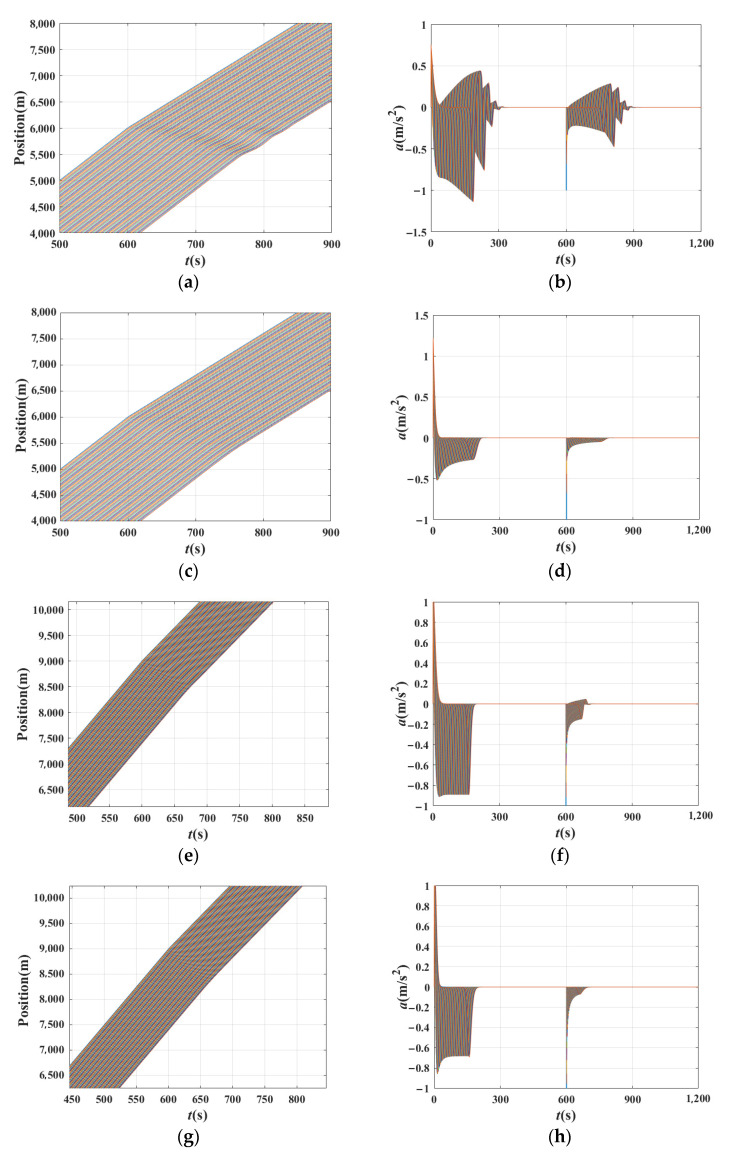
Time–position and acceleration changes in the traffic when basic model is the IDM. (**a**) Time–position of the traffic when *β*_1_ = 0, *β*_2_ = 0, T = 1.5 s; (**b**) Acceleration changes in the traffic when *β*_1_ = 0, *β*_2_ = 0, T = 1.5 s; (**c**) Time–position of the traffic when *β*_1_ = 0.4, *β*_2_ = 0, T = 1.5 s; (**d**) Acceleration changes in the traffic when *β*_1_ = 0.4, *β*_2_ = 0, T = 1.5 s; (**e**) Time–position of the traffic when *β*_1_ = 0.3, *β*_2_ = 0, T = 0.6 s; (**f**) Acceleration changes in the traffic when *β*_1_ = 0.3, *β*_2_ = 0.0, T = 0.6 s; (**g**) Time–position of the traffic when *β*_1_ = 0.3, *β*_2_ = 0.2, T = 0.6 s; (**h**) Acceleration changes in the traffic when *β*_1_ = 0.3, *β*_2_ = 0.2, T = 0.6 s.

Figure 6 shows time–position and acceleration changes in the traffic when the basic model is the IDM in experiment 1. Figure 6a,b,e,f shows that the disturbance is amplified when *β*_1_ and *β*_2_ are equal to 0. It reveals that the platoon tends to deviate from equilibrium in the wake of propagation of the perturbation, causing the instability of the traffic flow. In Figure 6c,d,g,h, the disturbance is diminished and the platoon is stable. This means that the traffic stability improved with the increase in *β*_1_ or *β*_2_, which is also consistent with the analytical results.

**Figure 7:**
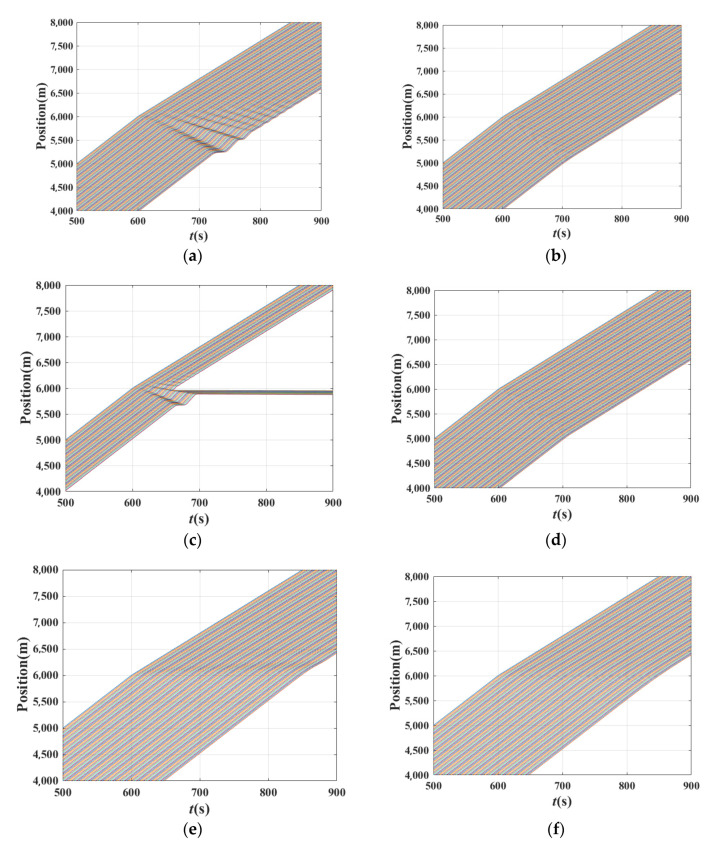
Time–position of the traffic when the basic model is the FVD, OV mode, and ACC model. (**a**) Time–position of the traffic when basic model is the FVD and *β*_1_ = 0, *β*_2_ = 0, ∆*x* = 20 m; (**b**) Time–position of the traffic when basic model is the FVD and *β*_1_ = 0, *β*_2_ = 0.8, ∆*x* = 20 m; (**c**) Time–position of the traffic when basic model is the OV model and *β*_1_ = 0., *β*_2_ = 0, ∆*x* = 20 m; (**d**) Time–position of the traffic when basic model is the OV model and *β*_1_ = 0.8, *β*_2_ = 0, ∆*x* = 20 m; (**e**) Time–position of the traffic when basic model is the ACC model and *β*_1_ = 0.0, *β*_2_ = 0, *t_hw_* = 2.5 s ACC; (**f**) Time–position of the traffic when basic model is the ACC model and *β*_1_ = 0.8, *β*_2_ = 0.0, *t_hw_* = 2.5 s.

**Figure 8:**
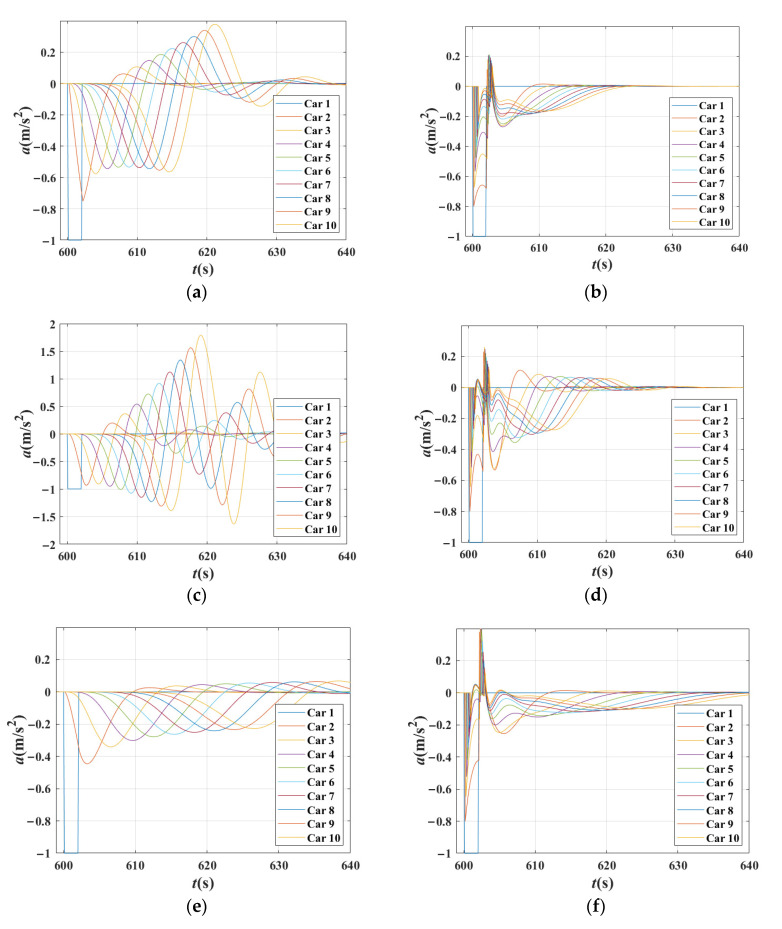
Acceleration changes in the traffic when the basic model is the FVD, OV, and ACC model. (**a**) Acceleration changes in the traffic when the basic model is the FVD and *β*_1_ = 0, *β*_2_ = 0, ∆*x* = 20 m; (**b**) Acceleration changes in the traffic when the basic model is the FVD and *β*_1_ = 0, *β*_2_ = 0.8, ∆*x* = 20 m; (**c**) Acceleration changes in the traffic when the basic model is the OV model and *β*_1_ = 0., *β*_2_ = 0, ∆*x* = 20 m; (**d**) Acceleration changes in the traffic when the basic model is the OV model and *β*_1_ = 0.8, *β*_2_ = 0, ∆*x* = 20 m; (**e**) Acceleration changes in the traffic when the basic model is the ACC model and *β*_1_ = 0.0, *β*_2_ = 0, *t_hw_* = 2.5 s ACC; (**f**) Acceleration changes in the traffic when the basic model is the ACC model and *β*_1_ = 0.8, *β*_2_ = 0.0, *t_hw_* = 2.5 s.

Figure 7 shows the time–position of the traffic when the basic model is the FVD, OV, and ACC model in experiment 1, respectively. Figure 8 shows the 1st~10th vehicles as examples to observe the transmission of disturbance between vehicles, helping to distinguish the traffic state. Figure 7a,c,e presents the shock waves that appeared in the traffic after the leading vehicle suffered from a small disturbance. In Figure 8a,c,e, the fluctuation of vehicles amplified along with the platoon. Comparing Figure 7a,c,e with Figure 8a,c,e, the instability of the platoon can be seen. While observing Figure 7b,d,f, it can be seen that the shock wave disappeared under these circumstances. This, combined with Figure 8b,d,f, shows the fluctuation of vehicles alleviated along with the platoon. Consequently, the traffic is stable under these conditions. These values are also consistent with the analytical results. Readers should note that in Figure 7c, the OV model has failed because of the instability, while vehicles could run normally under the BDVIS in Figure 7d.

In the traditional environment, vehicles make decisions depending on the moving information of the near preceding vehicle. In the connected environment, vehicles could receive accurate and abundant information from preceding and following vehicles. When a disturbance appeared, vehicles who followed the proposed structures could decide to respond to the perturbation in advance and coordinate with the movement of the following vehicle at the same time. However, in the traditional environment, vehicles obey the original car-following model, which neither foresees the intention of the preceding vehicle nor considers the movement of the vehicle followed. As a result, the proposed structures have a better performance in maintaining the string stability of the platoon. The theoretical and simulation results indicated that the BDVIS improves the traffic flow stability. The stability can be further improved by increasing the proportion of the sum of *β*_1_ and *β*_2_.

### 4.2. Sensitivity Analysis of β_1_ and β_2_ Based on the BDVIS

*β*_1_ and *β*_2_ represent the degree of considering the preceding and following vehicle, respectively. The moving direction of the traffic flow means that these two parameters have different effects on the vehicle. The later experiment is to explore the differences between *β*_1_ and *β*_2_ in the effect of traffic flow.

#### 4.2.1. The Disturbance Influence Time in Different Combination of *β*_1_ and *β*_2_

In experiment 2, the conditions *β*_1_ + *β*_2_ < 1 and *β*_1_ > *β*_2_ wre explored under the setting of *T* = 1.5 s. The mean DIT of 99 vehicles (except for the 1st vehicle) was calculated and drawn.

**Figure 9:**
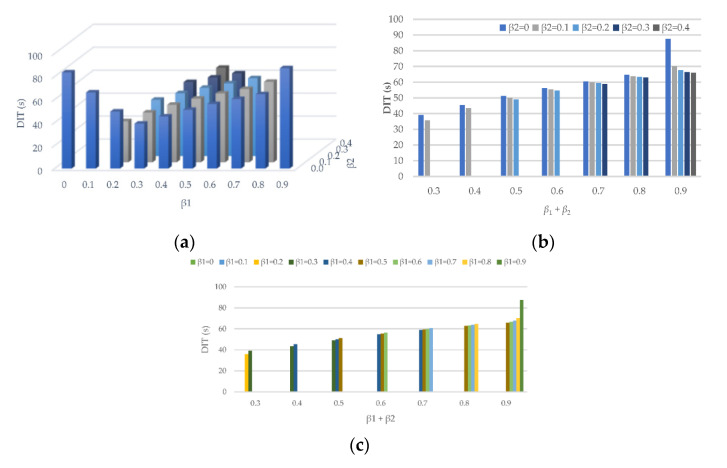
Mean value of DIT for 99 vehicles under the different combinations of *β*_1_ and *β*_2_ (s). (**a**) The relationship between DIT and *β*_1_ and *β*_2_; (**b**) The relationship between DIT and *β*_2_ when *β*_1_ + *β*_2_ is fixed; (**c**) The relationship between DIT and *β*_1_ when *β*_1_ + *β*_2_ is fixed.

From Figure 9a, it can be found that when *β*_2_ = 0, the DIT of vehicles decreases and then increases with the increase in *β*_1_. In other circumstances, the DIT of vehicles increases with the increase in either *β*_1_ or *β*_2_. The reason is that the traffic is unstable when *β*_1_ + *β*_2_ is small. If traffic is stable, any of the parameters extends the influence time. This reveals that neither *β*_1_ or *β*_2_ is the biggest or the best for the traffic. Figure 9b,c explores the influence of *β*_1_ and *β*_2_ when *β*_1_ + *β*_2_ is fixed. It can be seen, with the increase in *β*_2_, that the DIT decreased a little. As a consequence, the *β*_2_ presents the benefit of reducing DIT, while the *β*_1_ has the opposite influence.

#### 4.2.2. The Characteristics of Acceleration Curves in Different Combinations of *β*_1_ and *β*_2_

To investigate the reason why the *β*_1_ and *β*_2_ have different effects on the DIT, experiment 3 is conducted to analyze the characteristics of acceleration curves of each vehicle in the platoon under the different combinations of *β*_1_ and *β*_2_. Figures show the 1st~10th vehicles as examples to observe the transmission of disturbance between vehicles.

**Figure 10:**
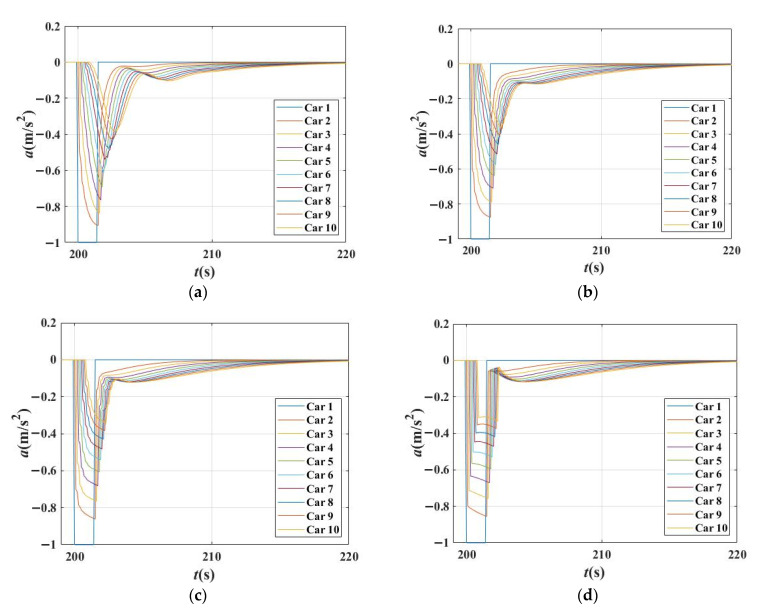
Acceleration changes for each vehicle in the platoon in different scenarios when *β*_1_ + *β*_2_ = 0.8 and time gap T = 1.5 s in IDM. (**a**) Acceleration changes for each vehicle in the platoon when *β*_1_ = 0.5, *β*_2_ = 0.3; (**b**) Acceleration changes for each vehicle in the platoon when *β*_1_ = 0.6, *β*_2_ = 0.2; (**c**) Acceleration changes for each vehicle in the platoon when *β*_1_ = 0.7, *β*_2_ = 0.1; (**d**) Acceleration changes for each vehicle in the platoon when *β*_1_ = 0.8, *β*_2_ = 0.

Figure 10 shows the acceleration changes for each vehicle in a platoon in four scenarios with different combinations of *β*_1_, *β*_2_,and *β*_1_ + *β*_2_ = 0.8. Figure 10 verifies the analysis results above; that the stability of these four cases is almost the same. However, the acceleration curves are not smooth in Figure 10a–c, especially in Figure 10b,c, in which there are more tiny fluctuations appearing in the curves. Therefore, although the stability of traffic is nearly the same in these circumstances, the reactions of vehicles differ.

**Figure 11:**
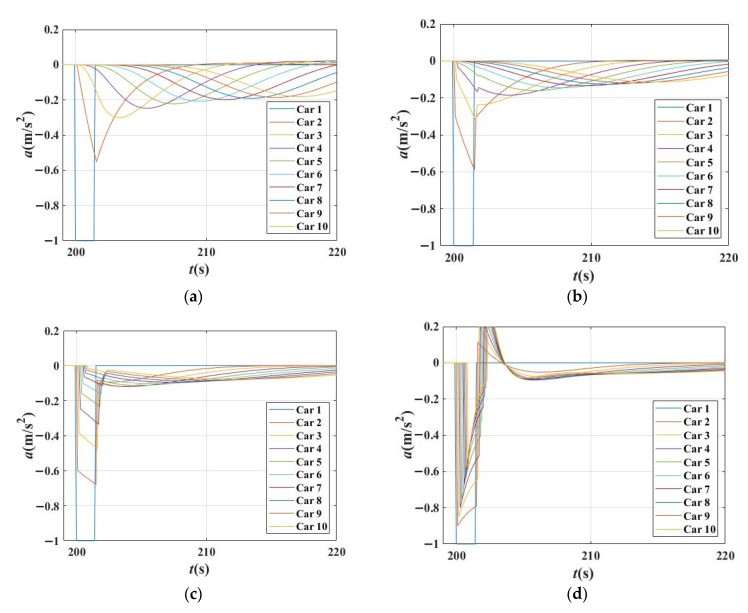
Acceleration changes for each vehicle in the platoon in different scenarios when time gap T = 1.5 s in IDM. (**a**) Acceleration changes for each vehicle in the platoon when *β*_1_ = 0, *β*_2_ = 0 (degenerated to IDM); (**b**) Acceleration changes for each vehicle in the platoon when *β*_1_ = 0.3, *β*_2_ = 0; (**c**) Acceleration changes for each vehicle in the platoon when *β*_1_ = 0.6, *β*_2_ = 0; (**d**) Acceleration changes for each vehicle in the platoon when *β*_1_ = 0.9, *β*_2_ = 0.

Figure 11 shows the acceleration changes for each vehicle in a platoon in four scenarios with *β*_1_ = 0, *β*_1_ = 0.3, *β*_1_ = 0.6 and *β*_1_ = 0.9, respectively. It can be seen that the traffic is unstable in Figure 11a,b, and stable in Figure 11c,d. With the increase in *β*_1_, the acceleration curves of vehicles became unsmooth, and more corners appeared. The shapes of these acceleration lines are close to the broken lines, and the acceleration change point appeared earlier. This reveals that the acceleration curves changed more rapidly and strongly with the increase in *β*_1_. The reason is that the vehicle responds quickly and strongly when it pays more attention to preceding vehicles.

**Figure 12:**
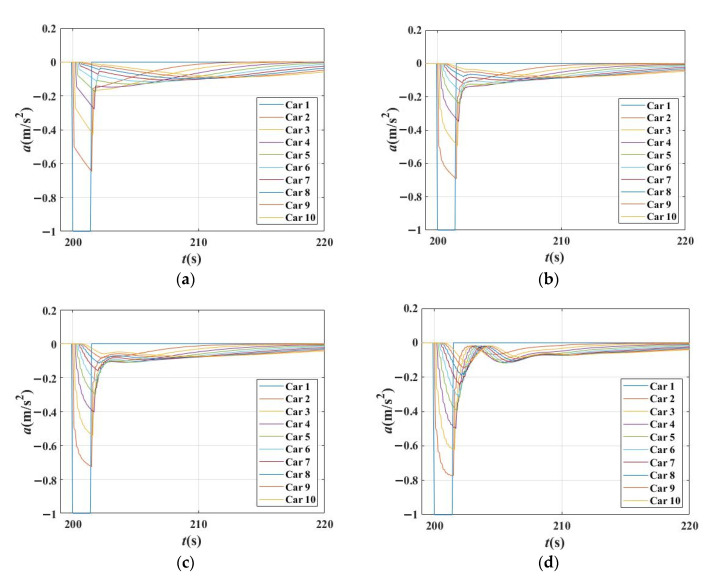
Relationship between the acceleration changes with β_2_ when time gap T = 1.5 s in IDM. (**a**) Acceleration changes for each vehicle in platoon when *β*_1_ = 0.5, *β*_2_ = 0; (**b**) Acceleration changes for each vehicle in platoon when *β*_1_ = 0.5, *β*_2_ = 0.2; (**c**) Acceleration changes for each vehicle in platoon when *β*_1_ = 0.5, *β*_2_ = 0.3; (**d**) Acceleration changes for each vehicle in platoon when *β*_1_ = 0.5, *β*_2_ = 0.4.

Figure 12 shows the relationship between the acceleration changes with *β*_2_. Under the settings that *β*_1_ = 0.5. It can be seen, when *β*_1_ is fixed, more tiny fluctuations appeared in the curves with the increase in *β*_2_. The acceleration curves are gentler, but the rough shape of these curves changed little. The smoothing effect of the *β*_2_ is achieved by increasing tiny fluctuations to reduce the large abrupt accelerate or decelerate.

Consequently, it can be concluded that when *β*_1_ increases, the acceleration curves change more rapidly and strongly, and the shape of acceleration curves change greatly. When *β*_2_ increases, the tiny fluctuation increased and the acceleration curves become more softly.

## 5. Conclusions

This paper proposes a bidirectional vehicles information structure (BDVIS) by making use of the acceleration information of one preceding vehicle and one following vehicle in a CV environment. Then, we deduce the derived multiple vehicles information structure (DMVIS), making use of the historical movement information of multiple vehicles, except for the acceleration information of near vehicles. Next, the paper embeds the IDM, FVD, OV, and ACC model into the framework, using linear stability theory to investigate the stability condition of two structures. Lastly, we conduct the simulations to verify the effects, and explore traffic response properties with different proportions and combinations of forward-looking or backward-looking terms. In addition, we define a disturbance influence time (DIT) to describe the acceleration fluctuation time influenced by the perturbation for each vehicle.

The following conclusions can be made:

(1) The theoretical and simulation results indicated the BDVIS and the DMVIS improve the traffic flow stability. The stability can be further improved by increasing the proportion of the acceleration of the preceding or following vehicles.
(2) If vehicles are on the two sides of the platoon, the BDVIS is better for improving string stability. If vehicles are in the middle section of the platoon and are able to obtain the multiple steps of historical information, DMVIS is a better option.
(3) The preceding and following proportions *β*_1_ and *β*_2_ have different influences on traffic response properties. The *β*_2_ has the benefit of reducing DIT, but the *β*_1_ extends DIT.
(4) If traffic is stable, any of the parameters extends the influence time. This reveals that neither *β*_1_ or *β*_2_ is the biggest and the best for the traffic.

In the future, the other effects of the *β*_1_ and *β*_2_, as well as the best combination of these parameters should be investigated to supplement the study for better implementation in the CV environment.

## Figures and Tables

**Table 1:** The introduction of the four car-following models.

Model	Formulation	*f_s_, f_v_, f*_∆*v*_	Parameters
IDM [39,40,41]	$a_{n}(t)=a\left[1-{\left(\frac{v_{n}(t)}{v_{0}}\right)}^{\delta }-{\left(\frac{s^{*}(v_{n}(t),\Delta v_{n,n-1}(t))}{s_{n}(t)}\right)}^{2}\right]$ $s^{*}(v_{n}(t),\Delta v_{n,n-1}(t))=s_{0}^{\left(n\right)}+T_{n}v_{n}(t)+\frac{v_{n}(t)\Delta v_{n,n-1}(t)}{2\sqrt{ab}}$	$f_{s}=\frac{2as^{*}{}^{2}}{s^{3}}$ $f_{v}=-\frac{4av^{3}}{v_{0}{}^{4}}-\frac{2aTs^{*}}{s^{2}}$ $f_{\Delta v}=-\frac{\sqrt{a}vs^{*}}{s^{2}\sqrt{b}}$	$a=1\;m/s^{2}$ $b=2\;m/s^{2}$ $v_{d}=120\;km/h$ $s_{0}=2\;m$ δ=4
FVD [42,43]	$\begin{array}{l} a=\lambda_{1}[V_{1}+V_{2}\tanh(C_{1}(s-l)-C_{2})-\\ v_{n}(t)]+\lambda_{2}\Delta v \end{array}$	$f_{s}=\lambda_{1}V_{2}C_{1}(1-\tanh^{2}\left[C_{1}(s-l)-C_{2}\right])$ $f_{v}=-\lambda_{1}$ $f_{\Delta v}=\lambda_{2}$	$\lambda_{1}=0.41\;s^{-1}$ $\lambda_{1}=0.4\;s^{-1}$ $V_{1}=6.75\;m/s$ $V_{2}=7.91\;m/s$ $C_{1}=0.13\;m^{-1}$ $C_{2}=1.75$ L=5 m
OV [42]	$a=\lambda_{1}\left[V_{1}+V_{2}\tanh\left[C_{1}(s-l)-C_{2}\right]-v_{n}(t)\right]$	$f_{s}=\lambda_{1}V_{2}C_{1}(1-\tanh^{2}\left[C_{1}(s-l)-C_{2}\right])$ $f_{v}=-\lambda_{1}$ $f_{\Delta v}=0$	$\lambda_{1}=0.85\;s^{-1}$ $V_{1}=6.75\;m/s$ $V_{2}=7.91\;m/s$ $C_{1}=0.13\;m^{-1}$ $C_{2}=1.75$ L=5 m
ACC [44]	$a=k_{1}(x_{k-1}-x_{k}-t_{hw}v_{k})+k_{2}(v_{k-1}-v_{k})$	$f_{s}=k_{1}$ $f_{v}=-k_{1}t_{hw}$ $f_{\Delta v}=k_{2}$	$k_{1}=0.23\;s^{-2}$ $k_{2}=0.07\;s^{-1}$

**Table 2:** The introduction of the experiments and their parameter settings.

Experiment	Basic Model	Parameter Settings			Characteristics
		*T/S/t_hw_*	*β*_1_	*β*_2_	
1	IDM	*T* = 1.5	0	0	unstable
			0.4	0	stable
		*T* = 0.6	0.3	0	unstable
			0.3	0.2	stable
	FVD	*S* = 20	0.0	0	unstable
			0.8	0	stable
	OV	*S* = 20	0.0	0	unstable
			0.8	0	stable
	ACC	*t_hw_* = 2.5	0.0	0	unstable
			0.8	0	stable
2	IDM	*T* = 1.5	0~0.4	0~0.9	*β*_1_ + *β*_2_ < 1, *β*_1_ > *β*_2_
3	IDM	*T* = 1.5	0.5	0.3	*β*_1_ + *β*_2_ = 0.8
			0.6	0.2	
			0.7	0.1	
			0.8	0	
	IDM	*T* = 1.5	0.0	0	*β*_2_ fixed
			0.3	0	
			0.6	0	
			0.9	0	
	IDM	*T* = 1.5	0.5	0	*β*_1_ fixed
			0.5	0.2	
			0.5	0.3	
			0.5	0.4	

## Data Availability

Not applicable.
